# Effects of Defatting Methods on the Physicochemical Properties of Proteins Extracted from *Hermetia illucens* Larvae

**DOI:** 10.3390/foods11101400

**Published:** 2022-05-12

**Authors:** Tae-Kyung Kim, Jae-Hoon Lee, Hae In Yong, Min-Cheoul Kang, Ji Yoon Cha, Ji Yeon Chun, Yun-Sang Choi

**Affiliations:** 1 Research Group of Food Processing, Korea Food Research Institute, Wanju 55365, Korea; kimtaekyung@kfri.re.kr (T.-K.K.); leejaehoon@kfri.re.kr (J.-H.L.); awsm_y@kfri.re.kr (H.I.Y.); mckang@kfri.re.kr (M.-C.K.); chajiyoon@kfri.re.kr (J.Y.C.); 2 Department of Food Bioengineering, Jeju National University, Jeju 63243, Korea; chunjiyeon@jejunu.ac.kr

**Keywords:** insect protein, cold pressure, protein characteristics, functional properties

## Abstract

In this study, we investigated the effects of various defatting methods, including organic solvent (aqueous, acetone, ethanol, and hexane) extraction and physical (cold pressure) extraction, on the nutritional, physicochemical, and functional properties of proteins extracted from *Hermetia illucens* larvae. The total essential amino acid contents were higher with cold pressure protein extraction than other treatments. The surface hydrophobicity with cold pressure treatment was the lowest, and there were no significant differences among the other treatments. The protein solubility after defatting with organic solvent was higher than for other treatments. The nonreduced protein band at 50 kDa of the defatted protein prepared using organic solvent was fainter than in the cold pressure treatment. The cold pressure-defatted protein showed the highest emulsifying capacity, and the water extracted protein showed the lowest emulsifying capacity. Although organic solvents may be efficient for defatting proteins extracted from insects, organic solvents have detrimental effects on the human body. In addition, the organic solvent extraction method requires a considerable amount of time for lipid extraction. Based on our results, using cold pressure protein extraction on edible insect proteins is ecofriendly and economical due to the reduced degreasing time and its potential industrial applications.

## 1. Introduction

Globally, the insect industry has recently been in the spotlight because of it has a very high potential for development [1]. Insects are already being used as a substitute for meat in many countries and insects can be used as a source of high-quality protein [2,3]. Insect-derived protein is easier to digest and absorb because it has a lower molecular weight than conventional meat [4]. However, the use of insects as food is limited because of the stigma of consuming them in Western society [2,5,6]. Therefore, converting edible insects into a powder or extract form is necessary to increase marketability and consumer acceptance [7]. Several studies have reported that powder or extract proteins derived from dried edible insects can be added to nutritionally enhance food [2,8,9,10]. However, lipid oxidation caused by 20–30 g lipid/100 g edible insects can affect the rancid odor of products [11]. Thus, a defatting process is required to remove edible insect lipids.

Organic solvents, such as ethanol, acetone, and hexane, have been used to remove fat components [11]. Mishyna et al. [12] reported that the protein extracted from edible insects used n-hexane as the defatting solvent to improve functional properties. Hexane is widely used in the food industry as an extraction solvent; however, residual hexane can be problematic. Zhao et al. [13] also defatted yellow mealworm protein using ethanol as a solvent. However, this use of organic solvents is costly and results in environmental or health problems, owing to residual solvents [14]. Despite these harmful effects, organic solvent-based defatting has only minimal effects on the functional properties of proteins [1,4]. Therefore, alternative defatting methods need to be developed. 

Cold pressure extraction is an extraction method that does not require heat or chemical treatment to obtain natural and safe products [14]. Cold pressure extraction is used to extract or remove vegetable oil and can inhibit protein denaturation by extracting lipids at controlled temperatures to inhibit lipid oxidation [14,15]. The use of cold pressure instead of degreasing using organic solvents should suppress the denaturation of insect proteins and thereby improve their functional properties.

Therefore, the objective of this study was to investigate the physicochemical properties and rheological properties of defatted proteins from *Hermetia illucens* larvae. Furthermore, we attempted to establish an ecofriendly protein extraction process for insects by defatting using cold pressure extraction without an organic solvent.

## 2. Materials and Methods

### 2.1. Materials

Ten kilograms of frozen *H. illucens* at the second instar stage were obtained in triplicate from a Real Nature Farm (Jeju, Korea). The larvae were fed on formulas of feed mixtures obtained from bio-waste and grown in cement boxes, and starved before freezing at −20 °C. Obtained frozen larvae were stored at −20 °C and defatting was carried out within a week after obtained frozen larvae. Acetone, ethanol, and hexane were obtained from Sigma-Aldrich Chemical Co. (St. Louis, MO, USA). 

### 2.2. Defatting, Processing, and Protein Extraction

All extraction processes were conducted on frozen and ground larvae. Chemical defatting processes were performed as described by Kim et al. [16]. For defatting, ground larvae (200 g) and a 99% organic solvent (1000 mL acetone, ethanol, or n-hexane) were stirred for 1 h at 20 °C. After discarding the organic solvent that contained insect fat, the same process was repeated five times until a clear solvent was obtained. The residual solvent was volatilized in a fume hood for 12 h at 20 °C. Cold pressure (Cold press-30; National Engineering, Goyang, Korea) was used for defatting at a feed rate of 4.18879 rad/s with temperatures of 80, 80, and 70 °C for the upper, middle, and lower layers, respectively. The oil cake thickness of cold pressure was fixed at 0.5 mm. Extracted fat components were discarded, and the residual sample was obtained to extract protein (46.2 °C). For protein extraction, 100 g defatted sample and 200 mL distilled water were homogenized at 1047.197551 rad/s for 2 min and centrifuged at 15,000× *g* for 30 min. The supernatant was filtered using a 500 μm pore-sized sieve, frozen at −20 °C, then freeze-dried using a freeze-dryer (Ilshinbiobase Co., Dongducheon, Korea). A water extract group was used as the control, and protein extraction was performed similarly to the treatment groups but without defatting. All extracted protein powder was stored at −20 °C before the experiments within a week.

### 2.3. Amino Acid and Essential Amino Acid Index

The amino acid composition of the protein powder extracted from larvae was determined according to Kim et al. [17]. An L-8800 amino acid analyzer (Hitachi, Tokyo, Japan; ion-exchange resin column (4.6 mm inner diameter × 60 mm)) was used to determine the amino acid composition. The essential amino acid index was measured by calculating the essential amino acid index (EAAI; FAO/WHO/UNU, 1985).

### 2.4. Surface Hydrophobicity

The surface hydrophobicity of the defatted insect proteins was estimated using the method of Chelh et al. [18]. Insect powder (0.5 g) was dissolved in 100 mL phosphate buffer (0.025 M, pH 7.4, 4 °C), 1 mL of the sample, and 200 μL of 0.1 mg/100 mL bromophenol blue (Bio-Rad Laboratories, Hercules, CA, USA). This was mixed and reacted at 20 °C for 10 min. A mixture of 100 mL phosphate buffer and 200 μL bromophenol blue was prepared as a blank. All reacted samples and blanks were centrifuged at 19,613.3 m/s^2^ for 15 min. The absorbance of 10-fold diluted solution in phosphate buffer was estimated at 595 nm using an Optizen 2120 UV plus UV/VIS spectrophotometer (Mecasys Co. Ltd., Daejeon, Korea). Bromophenol blue-bound protein (μg), which can show surface hydrophobicity, was calculated using the following formula:

(1) $$\mathrm{Bromophenol}\;\mathrm{blue}\;\mathrm{bound}\;(\mu g)=\frac{\mathrm{absorbance}\;\mathrm{of}\;\mathrm{control}\;\mathrm{at}\;595\;\mathrm{nm}\;-\;\mathrm{absorbance}\;\mathrm{of}\;\mathrm{sample}\;\mathrm{at}\;595\;\mathrm{nm}\;}{\mathrm{absorbance}\;\mathrm{of}\;\mathrm{control}\;\mathrm{at}\;595\;\mathrm{nm}}\;\times \;200\;\mu g$$

### 2.5. Protein Solubility

The protein solubility of the proteins extracted from larvae was determined following the methods of Kim et al. (2019). Briefly, freeze-dried protein from larvae was diluted with distilled water (pH 6.86), and the concentration was adjusted to 10 mg/mL. Then, the protein solubility of the extract was measured using Bradford reagent (Sigma-Aldrich, St. Louis, MO, USA).

### 2.6. Sodium Dodecyl Sulfate–Polyacrylamide Gel Electrophoresis (SDS-PAGE)

The molecular weight distribution of insect proteins was estimated using SDS-PAGE [19]. After adjusting the protein concentration to 1 mg/mL in sample buffer, 20 μL of solution was poured into Mini-PROTEIN TGX Gels (Bio-Rad Laboratories, Hercules, CA, USA) after heating at 100 °C for 5 min. Precision Plus Protein dual-color standards (Bio-Rad Laboratories) were used as standard markers. After running at 80 mA, protein bands were stained with Coomassie Brilliant Blue R 250 (Bio-Rad Laboratories). Samples were reduced using 2-mercaptoethanol.

### 2.7. pH Measurements

The pH of protein extracted from defatted *H. illucens* larvae was measured using a model 340 pH meter (Mettler-Toledo GmbH, Schwerzenbach, Switzerland), and pH 4, 7, and 10 buffers (Mettler-Toledo GmbH) were used as pH standard buffers.

### 2.8. Color Measurements

The color values of insect protein solutions were estimated using a CR-410 colorimeter (Minolta, Tokyo, Japan) attached to a CR-A50 granular attachment model (Minolta, Tokyo, Japan) according to the manufacturer’s instructions. Color values were presented using the International Commission on Illumination (CIE) L* a* b*color values. The illumination source was D65, and the observer degree was 2. The instrument was calibrated using a calibration plate (Y = 87.1, x = 0.3166, y = 0.3338). According to the CIE76 standard, the color difference (∆E) was calculated, and the water extract was used as the standard. 

### 2.9. Foam Capacity and Stability

After adjusting the protein concentration to 1 mg/mL in distilled water, foaming capacity and foam were determined according to the method of Mishyna et al. [12]. Ten milliliters of the sample were poured into a 50 mL conical tube and homogenized at 1256.637061 rad/s for 2 min. To estimate the foaming capacity, the foam volume was immediately compared with the initial volume of the solution. Changes in foam volume were recorded after 2, 5, 10, 20, 30, and 60 min and were calculated as mL/100 mL to estimate the foam stability. 

### 2.10. Emulsion Capacity and Emulsion Stability

The emulsion capacity and emulsion stability were determined as previously described by Pearce and Kinsella [20] with modifications. For emulsion capacity, 1 mL olive oil was added to 10 mL protein that was extracted from defatted *H. illucens* larvae, and homogenized at 1884.955592 rad/s for 2 min. The homogenized sample was allowed to stand for 10 min, and the emulsified layer and initial volume were compared and expressed as a percentage.

To determine the emulsion stability of the protein extracted from defatted *H. illucens* larvae, a 50 μL emulsion was added to 10 mL SDS solution (0.3 mg/100 mL) and inverted several times. Changes in the absorption of each emulsion were detected at a wavelength of 500 nm. The emulsion stability was calculated by dividing the absorbance after the interval time by the initial absorbance and multiplying by 100. The interval times were 10, 20, 30, 40, 50, 60, 90, and 120 min.

### 2.11. Statistical Analysis

Statistical Package for Social Sciences 20 software (SPSS Inc., Chicago, IL, USA) was used to analyze the data statistically. A one-way analysis of variance with the Duncan’s range test was performed (*p* < 0.05). Fixed effects were considered when the effects of defatting methods were compared. All experiments were performed in triplicate, and protein extraction from *H. illucens* larvae was evaluated for each replicate. Replicates were considered as random effects.

## 3. Results and Discussion

### 3.1. Amino Acid Composition and Essential Amino Acid Index

When calculating the crude protein content of insects, the conventional nitrogen conversion factor (6.25) should be modified because of nonprotein nitrogen compounds, such as chitin [21]. Protein extraction could enhance the factor value by decreasing chitin content, and different amino acid components have been observed in previous studies by using different defatting methods and insect species [1,17]. Therefore, the amino acid composition should be estimated to obtain the nutritional value in insects. The amino acid composition of the protein extracted from *H. illucens* larvae using various defatting methods is shown in Table 1. Among essential amino acids, the contents of His, Ile, Leu, Phe + Tyr, and Val were highest when the cold pressure protein extraction method was used (*p* < 0.05), whereas that of Lys was highest when the water extraction method was used (*p* < 0.05). In addition, the total essential amino acid contents were highest when cold pressure was used for the defatting proteins that were extracted from *H. illucens* larvae (*p* < 0.05).

The total essential amino acid was lowest in the hexane treatment (*p* < 0.05). Essential amino acids cannot be synthesized by the organism and must be obtained through the diet. Thus, essential amino acid intake is important for human nutrition, and their recommended intake has been investigated and widely used [22]. For nonessential amino acids, the contents of Als, Arg, Asp, and Gly were higher in the cold pressure treatment group than in the group treated with organic solvent (*p* < 0.05). However, the total nonessential amino acid contents were highest in the group treated with hexane (*p* < 0.05). These different amino acid compositions may be related to the chemical or physical denaturation of insect proteins. Protein denaturation could affect protein solubility during drying, defatting, and extraction. The organic solvent, pH condition, or heating denatured the insect proteins [1,23]. These changes in protein structure can affect protein characteristics, and changes in protein solubility can affect amino acid profile [1]. However, according to Queiroz et al. [24], the unfolding temperature of extracted protein from *H. illucens* ranged from 110 to 140 °C. Therefore, protein cold pressure treatment might not denature proteins, or result in a small amount of protein being denatured by cold pressure instrument.

Although the protein contents in the extracts were similar, their nutritional value could differ, and cold pressure treatment yielded the highest nutritional value among all treatments (*p* < 0.05). Protein hydrophobicity depends on the hydrophobic amino acid composition exposed at the surface. The balance of hydrophilic or hydrophobic amino acids can affect protein functionality, such as foaming and emulsifying properties [23]. In this study, the composition of hydrophilic and hydrophobic amino acids in all treatments were compared, and the most similar ratio was observed under cold pressure treatments (data not shown). Therefore, cold pressure may have good nutritional and functional qualities. 

### 3.2. Surface Hydrophobicity, Protein Solubility, pH, and Color

Structural characteristics, protein solubility, and pH should be considered to improve protein techno-functionality [23]. The effects of the defatting method on protein characteristics, such as surface hydrophobicity, protein solubility, and pH, are shown in Table 2. The surface hydrophobicity was lowest for the samples treated with cold pressure (*p* < 0.05), and there were no significant differences among the other samples (*p* > 0.05). Buried hydrophobic residues can be exposed to protein denaturation, affecting the tension of the protein film [25]. Therefore, higher hydrophobicity can result in higher protein functionalities because of changes in the interfacial or surface tension [23]. However, if the surface hydrophobicity is greater than a certain degree, binding forces between proteins can be stronger, and unpredictable protein aggregation can occur [1]. Organic solvents can change the structural characteristics of the protein, affecting surface hydrophobicity and solubility [26]. Therefore, the protein solubility obtained after the defatting treatment with organic solvents was higher than the other treatments (*p* < 0.05). However, the water extract had similar surface hydrophobicity values when compared with the organic solvents treatments (*p* > 0.05). This may be because of insufficient defatting when compared with the other treatments. Kim et al. [1] also reported that the surface hydrophobicity of aqueous extracts was higher than the other methods because of the high-fat content in the extract.

Generally, protein functionality is increased away from the isoelectric point of a protein, which is typically close to 5.0 for insect proteins [12,23]. Therefore, changes in pH during extraction should be controlled, and differences in pH values could explain the different functions of the proteins [27]. Although there was a significant difference between cold pressure and the other methods (*p* < 0.05; Table 2), the difference was very small (0.02 points). Therefore, protein functionality differences among treatments caused by pH may be minimized.

Color pigments such as melanin can be affected by various conditions during the extraction of insect proteins [1]. In this study, CIE L*, CIE a*, and CIE b* values, as well as color differences of the hexane treatment, were higher than for the other treatments (*p* < 0.05). In a related study, a protein obtained from *Protaetia brevitarsis* was affected by organic solvents, and the highest color difference was also observed in the hexane treatment [1]. As shown in Table 2, the color difference for the hexane treatment only exceeded one, and that of the others was lower than one. The human eye can detect color differences when the color difference value between samples exceeds one [28]. Therefore, although some significant differences were detected in our statistical analysis of the treatments, consumers could not detect differences among treatments, except for the hexane treatment.

### 3.3. SDS-PAGE

**Figure 1:**
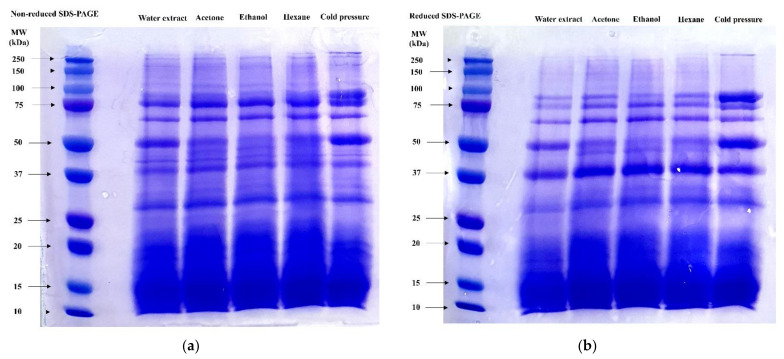
Effects of different defatting methods on sodium dodecyl sulfate-polyacrylamide gel electrophoresis (SDS-PAGE) of extracted nonreduced (**a**) and reduced (**b**) proteins from *Hermetia illucens* larvae.

The molecular weight distributions of insect proteins are presented in Figure 1. Because it was difficult to separate parts from tiny insects, the various proteins in insects, such as muscle tissues, organs, and hemolymph, were extracted without separation in this study [29]. Similar patterns of protein bands were observed but with varying intensities (Figure 1). When compared to the control, the protein band at 50 kDa was fainter for the nonreducing organic solvent and cold pressure treatment (Figure 1a). The interfacial films of proteins can be affected by protein molecular weight, and the formation of high-molecular-weight protein polymers could help achieve good protein functionalities [23]. Disulfide bonds can occur via protein denaturation, which can be observed through a reducing method using mercaptoethanol [19]. Figure 1b shows that all treatments had a stronger intensity at 37 kDa, and fainter bands were observed above 100 kDa after reduction. Protein resistance to denaturation may differ depending on the protein source and can be affected by chemical reagents, temperature, pH, and processing method [30]. Protein denaturation during extraction induces changes in protein solubility owing to structural changes [23]. According to the data shown in Table 1, the amino acid composition differed according to the defatting method used, and these different compositions may be correlated with variations in the molecular weight distributions of the proteins. Overall, high-molecular-weight proteins with high resistance to denaturation could be extracted after removing fat using cold pressure, and this could help enhance protein functionalities.

### 3.4. Foaming Capacity and Foam Stability

**Figure 2:**
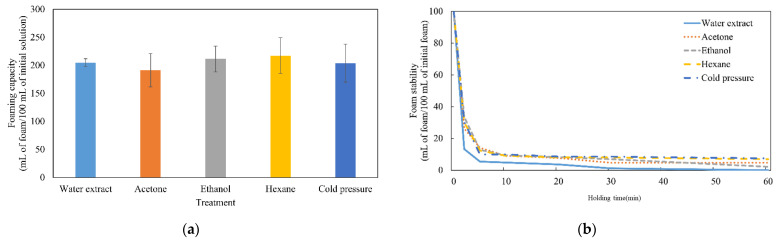
Effects of different defatting methods on foaming capacity (**a**) and foam stability (**b**) on the extracted protein solutions from *Hermetia illucens* larvae. Control and treatments (acetone, ethanol, hexane, cold pressure) are indicated using different line types and colors (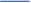, 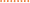, 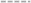, 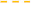, 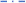).

Foaming properties were assessed to measure foaming capacity and foam stability. As shown in Figure 2a, there were no significant differences among treatments in foaming capacity (*p* > 0.05). Foaming capacity depends on protein solubility, surface flexibility, and hydrophobicity [12]. Although the protein solubility of the control and cold pressure groups was lower than that of the other groups, the large amount of high-molecular-weight proteins in these extracts could support the high foaming capacity, because thick films can physically hold their structure better than thin films [23]. However, the foams collapsed rapidly, and all treatments lost over 90 mL/100 mL of foam after 10 min (Figure 2b). Among treatments, the foam collapse speed of the water extract was the fastest (loss of >85 mL/100 mL of foam after 2 min; *p* < 0.05). Mishyna et al. [12] reported that the dominance of low-molecular-weight (5–30 kDa) components, such as proteins and polysaccharides, in insects could enhance foam stability. As shown in Figure 1, most protein bands were concentrated below 37 kDa. However, because the water extract was prepared without any defatting step, the remaining fat components may have inhibited the formation of a stable foam structure [1].

### 3.5. Emulsifying Capacity and Emulsion Stability

**Figure 3:**
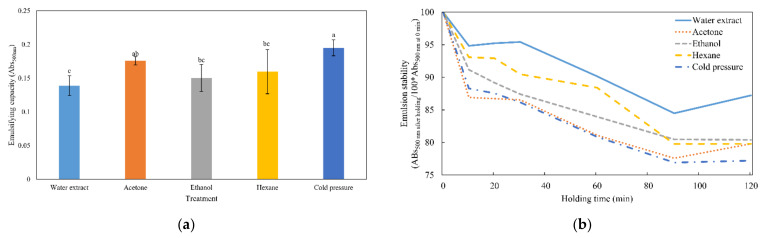
Effects of different defatting methods on the emulsifying capacity (**a**) and emulsion stability (**b**) of extracted proteins from Hermetia illucens larvae. Control and treatments (acetone, ethanol, hexane, cold pressure) are indicated using different line types and colors (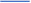, 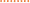, 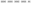, 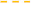, 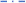). Different letters on top of columns indicated significant differences among treatments (*p* < 0.05).

Emulsifying capacity plays an important role in forming the frame and texture of food [17]. The emulsifying capacity and emulsion stability results are shown in Figure 3. Among the protein samples, cold pressure-defatted protein showed the highest emulsifying capacity (*p* < 0.05), and the water extract protein showed the lowest emulsifying capacity (*p* < 0.05). Mishyna et al. [12] reported that excessively hydrophobic proteins have strong binding forces between proteins and are unsuitable for emulsion formation. In addition, the cold pressure-defatted proteins had a lower surface hydrophobicity than the other defatted proteins and the water extract. Thus, the cold pressure-defatted proteins had the highest emulsifying capacity.

Furthermore, the electrophoresis results (Figure 1) showed there were no significant changes in the protein composition when different defatting methods were used. Damodaran [31] reported that low-molecular-weight proteins are more effective at forming emulsions. In addition, other studies have reported that low-molecular-weight emulsifiers show better emulsion capacity [32,33]. In this study, however, the abundance of low-molecular-weight proteins was high, and this dispersion may have affected the emulsifying properties of the solution. The emulsion stability results are shown in Figure 3b. At 120 min, protein samples that were defatted using acetone, ethanol, and hexane extraction and cold pressure treatment showed a lower emulsion stability than the protein samples subjected to water extraction. During the defatting process, the interaction between proteins is weakened, and the interfacial tension is decreased, reducing emulsion stability [34,35].

## 4. Conclusions

The effects of various defatting methods, such as organic solvent extraction (aqueous, acetone, ethanol, and hexane) and physical extraction (cold pressure) on the amino acid profiles, physicochemical characteristics, and functional properties of proteins extracted from *H. illucens* larvae were investigated. The cold pressure extraction method with nonorganic solvent extraction showed excellent characteristics in terms of the nutritional properties of insect protein extracts. The physicochemical characteristics and functional properties of the insect protein treatment group that underwent extraction with an organic solvent were high. However, there were no significant differences when compared with the cold pressure extraction group. Organic solvents can have detrimental effects on the human body when ingested. Thus, cold pressure could be used at the industrial level when extracting edible insect protein as an ecofriendly extraction method. In addition, the organic solvent extraction method requires more time for lipid extraction; however, the cold pressure method has the advantage of shortening the extraction time. Therefore, the cold pressure method could be used as an appropriate and effective defatting method. Based on these findings, this approach may have industrial applications as a defatting process in the preparation of edible insect proteins.

## Figures and Tables

**Table 1:** Effects of different defatting methods on amino acid profiles and essential amino acid index for proteins extracted from *Hermetia illucens* larvae.

	Water Extract	Acetone	Ethanol	Hexane	Cold Pressure	ReferenceValue ^1^
Essential amino acids						
His	44.87 ± 1.16 ^bc^	44.06 ± 0.01 ^c^	45.78 ± 0.23 ^b^	44.56 ± 0.47 ^bc^	56.02 ± 0.44 ^a^	15
**Ile**	27.17 ± 0.01 ^b^	26.89 ± 0.10 ^b^	26.63 ± 0.56 ^bc^	25.86 ± 0.12 ^c^	28.82 ± 0.64 ^a^	30
**Leu**	38.77 ± 0.57 ^b^	38.05 ± 0.45 ^bc^	36.80 ± 0.79 ^c^	36.84 ± 0.10 ^c^	41.16 ± 0.53 ^a^	59
**Lys**	95.09 ± 0.31 ^a^	87.93 ± 0.02 ^d^	88.65 ± 0.28 ^c^	91.86 ± 0.26 ^b^	86.48 ± 0.27 ^e^	45
**Met** + Cys	11.69 ± 0.06 ^c^	12.09 ± 0.02 ^b^	12.10 ± 0.01 ^b^	12.57 ± 0.03 ^a^	10.21 ± 0.06 ^d^	22
**Phe** + Tyr	99.50 ± 0.35 ^b^	98.27 ± 0.40 ^bc^	97.00 ± 0.40 ^c^	93.68 ± 1.40 ^d^	108.30 ± 0.13 ^a^	38
Thr	35.31 ± 0.18 ^c^	35.68 ± 0.17 ^c^	38.13 ± 0.58 ^a^	36.61 ± 0.03 ^b^	36.61 ± 0.11 ^b^	23
**Val**	37.02 ± 0.47 ^bc^	37.57 ± 0.34 ^b^	36.67 ± 0.20 ^c^	36.85 ± 0.04 ^bc^	42.08 ± 0.26 ^a^	39
Sum of EAA	389.4 ± 0.34 ^b^	380.51 ± 0.26 ^cd^	381.74 ± 1.29 ^c^	378.8 ± 0.69 ^d^	409.64 ± 1.95 ^a^	271
Nonessential amino acids						
Ala	123.81 ± 0.23 ^b^	123.98 ± 0.69 ^b^	117.3 ± 0.17 ^c^	123.17 ± 0.18 ^b^	135.78 ± 0.47 ^a^	
Arg	47.12 ± 0.09 ^d^	47.67 ± 0.21 ^cd^	49.15 ± 0.51 ^b^	47.94 ± 0.17 ^c^	52.58 ± 0.09 ^a^	
Asp	85.55 ± 0.36 ^c^	88.64 ± 0.71 ^b^	92.76 ± 0.11 ^a^	89.36 ± 0.07 ^b^	91.95 ± 0.09 ^a^	
Glu	185.79 ± 0.83 ^c^	191.50 ± 1.20 ^b^	196.44 ± 0.81 ^a^	193.12 ± 0.10 ^b^	155.88 ± 0.60 ^d^	
**Pro**	73.94 ± 1.52 ^a^	73.16 ± 3.38 ^a^	64.25 ± 0.67 ^b^	73.53 ± 0.60 ^a^	62.94 ± 3.09 ^b^	
**Gly**	48.86 ± 0.05 ^b^	49.34 ± 0.42 ^b^	50.47 ± 0.03 ^a^	49.04 ± 0.14 ^b^	52.11 ± 0.09 ^a^	
Ser	45.56 ± 0.36 ^b^	45.24 ± 0.84 ^b^	47.94 ± 0.8 ^a^	45.07 ± 0.37 ^b^	39.16 ± 0.02 ^c^	
Sum of nonessential amino acids	610.61 ± 0.34 ^c^	619.5 ± 0.26 ^ab^	618.27 ± 1.29 ^b^	621.21 ± 0.69 ^a^	590.37 ± 1.95 ^d^	
Essential amino acid index	1.26 ± 0.01 ^b^	1.25 ± 0.01 ^bc^	1.25 ± 0.01 ^bc^	1.24 ± 0.01 ^c^	1.31 ± 0.01 ^a^	1

All values are means ± standard deviations of three replicates (*n* = 3). The units for amino acids are mg amino acid/1 g protein. ^a–d^ Means within a row with different letters are significantly different (*p* < 0.05). Hydrophobic amino acids are presented in bold text. ^1^ Reference values of essential amino acid requirements for humans were from FAO/WHO/UNU (1985).

**Table 2:** Effects of different defatting methods on the surface hydrophobicity, protein solubility, pH, and color characteristics of an extracted protein solution from *Hermetia illucens* larvae.

	Water Extract	Acetone	Ethanol	Hexane	Cold Pressure
Surface hydrophobicity (Bromophenol blue bound, μg)	35.69 ± 3.79 ^a^	33.90 ± 6.84 ^a^	30.58 ± 6.47 ^a^	37.45 ± 2.80 ^a^	11.65 ± 1.75 ^b^
Protein solubility (mg/mL)	41.60 ± 0.17 ^d^	52.42 ± 0.74 ^a^	52.80 ± 0.78 ^a^	50.40 ± 0.29 ^b^	44.19 ± 0.02 ^c^
pH	7.14 ± 0.01 ^ab^	7.13 ± 0.01 ^b^	7.14 ± 0.01 ^ab^	7.14 ± 0.00 ^a^	7.12 ± 0.01 ^c^
CIE L*	16.67 ± 0.01 ^c^	16.37 ± 0.01 ^d^	16.83 ± 0.02 ^c^	18.01 ± 0.01 ^a^	17.24 ± 0.31 ^b^
CIE a*	1.86 ± 0.06 ^d^	1.82 ± 0.06 ^d^	1.95 ± 0.08 ^c^	2.22 ± 0.02 ^a^	2.09 ± 0.09 ^b^
CIE b*	4.07 ± 0.04 ^b^	3.98 ± 0.03 ^c^	3.85 ± 0.06 ^d^	4.14 ± 0.03 ^a^	3.87 ± 0.03 ^d^
Color difference (∆E)	- ^1^	0.32 ± 0.03 ^c^	0.31 ± 0.06 ^c^	1.39 ± 0.01 ^a^	0.65 ± 0.31 ^b^

All values are means ± standard deviations of three replicates (*n* = 3). ^a–d^ Means within a row with different letters are significantly different (*p* < 0.05). ^1^ The color value of water extract was used as a standard to compare color differences between other treatments, and the color difference was calculated according to the CIE76 ∆E formula (∆L^*2^ + ∆a^*2^ + ∆b^*2^)^1/2^.

## Data Availability

Data are contained within the article.
